# Influence of past breast feeding on pattern and severity of presentation of juvenile idiopathic arthritis

**DOI:** 10.1136/archdischild-2014-308117

**Published:** 2015-09-14

**Authors:** Kimme L Hyrich, Eileen Baildam, Hannah Pickford, Alice Chieng, Joyce E Davidson, Helen Foster, Janet Gardner-Medwin, Lucy R Wedderburn, Wendy Thomson

**Affiliations:** 1 Arthritis Research UK Centre for Epidemiology, Centre for Musculoskeletal Research, Institute of Inflammation and Repair, The University of Manchester, Manchester Academic Health Science Centre, Manchester, UK; 2 NIHR Manchester Musculoskeletal Biomedical Research Unit, Central Manchester NHS Foundation Trust, Manchester Academic Health Sciences Centre, Manchester, UK; 3 Department of Rheumatology, Alder Hey Children's NHS Foundation Trust Hospital, Liverpool, UK; 4 Department of Rheumatology, The Royal Manchester Children's Hospital, Central Manchester University Hospitals NHS Foundation Trust, Manchester, UK; 5 Department of Rheumatology, Royal Hospital for Sick Children, Greater Glasgow and Clyde Health Board and Royal Hospital for Sick Children, Edinburgh, UK; 6 Department of Rheumatology, Newcastle University and Great North Children's Hospital, Newcastle Hospitals NHS Foundation Trust, Newcastle upon Tyne, UK; 7 Department of Rheumatology, Glasgow University, Glasgow, UK; 8 Infection, Immunology, and Rheumatology Section UCL Institute of Child Health, University College London, London, UK; 9 Department of Rheumatology, Great Ormond Street Hospital NHS Foundation Trust, London, UK; 10 Centre for Adolescent Rheumatology at University College London, University College London Hospital, London, UK

**Keywords:** Rheumatology, Epidemiology, Infant Feeding

## Abstract

This analysis aimed to study the influence of breast feeding on the pattern and severity of juvenile idiopathic arthritis (JIA) at presentation. The association between ever versus never breast feeding and disease severity at onset was compared in 923 children with JIA recruited to the UK Childhood Arthritis Prospective Study at first presentation to rheumatology. Fifty six per cent of children were ever breast fed (median 3.7 months). Breastfed children reported a lower median age at onset, a lower Childhood Health Assessment Questionnaire (CHAQ), a measure of disease severity, lower parent general evaluation scores and lower pain at presentation. There was a trend towards a higher proportion of breastfed children with rheumatoid factor-negative polyarthritis, but lesser enthesitis-related and psoriatic arthritis. There was a statistically significant inverse association between breast feeding and high CHAQ, even after adjusting for differences in socioeconomic status (adjusted OR 0.61 (95% CI 0.39 to 0.95)). Further work to understand the reasons behind these associations is required.

**What is already known on this topic**

- The aetiology of juvenile idiopathic arthritis remains poorly understood.
- Research into environmental and genetic risk factors has been conflicting.
- Breast feeding may be associated with lesser occurrence or delayed onset of autoimmune diseases.

**What this study adds**

- Patterns of arthritis may differ between children ever versus never breast fed.
- Breast feeding may be associated with a milder onset of juvenile idiopathic arthritis.

## Introduction

Juvenile idiopathic arthritis (JIA) is a heterogeneous chronic inflammatory arthritis, classified by the International League Against Rheumatism (ILAR) into seven categories. The aetiology remains unknown.

There is interest in the association between breast feeding, immune system development and future development of autoimmune diseases. Breast milk is a rich source of immunological defences, which help to stimulate the infant's immature immune system, and may have an important role in the establishment of gut flora and defence against infection.

The potential relationship between breast feeding and susceptibility to JIA has been described in small case–control studies,^1–4^ although only one study has suggested an association between breast feeding and decreased susceptibility. Two studies found shorter^3^ or less frequent^1^ breast feeding among children who developed oligoarticular disease compared with controls, which was not confirmed in a third study.^2^ No study has looked at the severity or timing of JIA presentation. Our aim was to study the influence of exposure to, and duration of, breast feeding on pattern and severity of JIA at first presentation to rheumatology.

## Methods

This study included children in the Childhood Arthritis Prospective Study (CAPS) using methods, which have been detailed elsewhere.^5^ In brief, at presentation to paediatric rheumatology and at regular follow-up, demographic and clinical data are collected from the medical record, and families attend an interview to detail birth and health history, including whether the child was breast fed and for how long. CAPS was approved by the UK Research Ethics Committee, and written consent was obtained from all participants.

Baseline demographic and clinical characteristics were compared between children ever versus never breast fed using non-parametric descriptive statistics. Socioeconomic status (SES) was based on the patient's postcode, and classified using the 2007 Index of Multiple Deprivation (IMD), an England-specific location-based deprivation score, which takes into consideration a number of social indicators. Children were grouped into three national ranked categories. IMD scores are not comparable across the four countries in the UK, and therefore, SES data are only presented for children resident in England (80% of cohort). Characteristics were also analysed according to the duration of breast feeding using univariable logistic and linear regression models. An association between ever breast feeding and moderate-to-severe disease at presentation, defined using a Child Health Assessment Questionnaire (CHAQ) score >0.75^6^, was explored using a multivariable logistic regression model, adjusted for a priori selected covariates (onset age, gender, hospital, disease duration, ILAR category and IMD) as well as any variables significant in univariable analysis. Multiple imputation (25 iterations) was used to account for missing data.

## Results

To 05/2012, 1253 children had been recruited; 1015 (81%) attended for interview, and 923 (74%) answered the breastfeeding questions. Those without breastfeeding data were older (median 8.6 vs 6.4 years, p<0.001) with lower disease activity (median Physician Global Assessment (PGA) score 24/100 vs 29/100, p=0.01), but were otherwise similar.

Five hundred and thirteen (56%) children were reported to have ever breast fed; median duration 3.7 months, 14% ≥6 months, 5% ≥1 year.

There were many demographic and clinical differences between children ever versus never breast fed (table 1), including presentation at a younger age and lower deprivation scores. In general, disease activity and severity were also lower among those ever breast fed, with lower PGA and Juvenile Arthritis Disease Activity Scores (JADAS). Patient-reported outcomes (CHAQ including the proportion of children with CHAQ >0.75), pain and Parent General Evaluation (PGE) scores were all significantly lower in children ever breast fed. There was also a small difference noted across the distribution of ILAR categories with a higher proportion of polyarthritis and a lower proportion of enthesitis-related arthritis (ERA) and psoriatic arthritis (PsA) among those ever versus never breast fed. The proportion of children presenting with an oligoarticular pattern was identical in both groups. Dose–response relationships were also observed in the age of onset, ethnicity, IMD, PGA and the CHAQ score (table 2), but less evident in other measures.

**Table 1:** Demographic factors and disease characteristics at presentation in children with JIA ever versus never breast fed

Characteristic	Total cohort	Never breast fed	Ever breast fed	p Value (ever vs never breast fed)	Univariate analysis of association between covariate and CHAQ >0.75 (OR, 95% CI)
N (%)	923	410 (44)	513 (56)		
Female, n (%)*	604 (65)	262 (64)	342 (67)	0.4	0.87 (0.61 to 1.23)*
Caucasian, n (%)*	831 (90)	381 (93)	450 (88)	0.009	1.08 (0.65 to 1.80)*
Index of Multiple Deprivation category (n=737), n (%)*,†					
Low	143 (20)	37 (11)	106 (26)	<0.001	Ref
Medium	334 (45)	139 (43)	195 (47)		1.33 (0.86 to 2.06)
High	260 (35)	149 (46)	111 (27)		1.76 (1.13 to 2.74)
Referral hospital, n (%)*					
Liverpool	383 (42)	183 (45)	200 (39)	<0.001	Ref
Manchester	174 (19)	83 (20)	91 (18)		0.91 (0.60 to 1.38)
Glasgow	139 (15)	68 (16)	71 (14)		–
Newcastle	50 (5)	28 (7)	22 (4)		0.87 (0.45 to 1.68)
London	177 (19)	48 (12)	129 (25)		1.07 (0.71 to 1.62)
Age at symptom onset (years), median (IQR)*	6.4 (2.5, 11)	7.6 (3.2, 11)	5.7 (2.0, 10)	<0.001	0.97 (0.93 to 1.01)
Symptom duration at first visit (months), median (IQR)*	5.6 (2.9, 12)	5.6 (2.8, 13)	5.6 (2.9, 11)	0.6	1.00 (0.99 to 1.01)
Active joint count, median (IQR)	2 (1, 5)	2 (1, 5)	2 (1, 5)	0.6	–
Physician Global Assessment (100 mm VAS) (n=743), median (IQR)	29 (16, 53)	32 (18, 56)	28 (15, 50)	0.03	–
ESR (n=570), median (IQR)	20 (6, 50)	18 (6, 47)	22 (7, 53)	0.1	–
Parent global assessment (100 mm VAS) (n=656),median (IQR)	21 (5, 50)	39 (10, 64)	23 (5, 50)	<0.001	–
JADAS-71,‡ median (IQR), (n=340)*	11 (6–18)	13 (7–20)	10 (6–17)	0.02	1.10 (1.07 to 1.12)
Limited joint count, median (IQR)*	1 (1, 3)	1 (1, 3)	1 (1, 3)	0.3	1.09 (1.04 to 1.14)
Pain (100 mm VAS) (n=664), median (IQR)*	30 (8, 58)	29 (7, 54)	18 (4, 45)	<0.001	1.04 (1.03 to 1.05)
CHAQ score (n=674), median (IQR)	0.63 (0.13, 1.38)	0.88 (0.25, 1.63)	0.63 (0.13, 1.25)	<0.001	
% CHAQ >0.75 (n=674), n (%)	303 (45)	159 (54)	144 (38)	<0.001	
ILAR subtype, n (%)*					
Systemic	49 (5)	23 (6)	26 (5)	0.05	Ref
Oligoarthritis (persistent)	427 (46)	189 (46)	238 (46)		0.56 (0.27 to 1.17)
Oligoarthritis (extended)	59 (7)	28 (7)	31 (6)		1.19 (0.43 to 3.31)
Polyarthritis (RF−)	215 (23)	79 (19)	136 (27)		1.69 (0.73 to 3.79)
Polyarthritis (RF+)	27 (3)	11 (3)	16 (3)		2.36 (0.59 to 9.46)
Enthesitis related	52 (6)	32 (8)	20 (4)		0.78 (0.29 to 2.10)
Psoriatic	67 (7)	36 (8)	31 (6)		0.62 (0.24 to 1.57)
Undifferentiated	27 (3)	12 (3)	15 (3)		0.47 (0.14 to 1.60)
OR (95% CI) CHAQ >0.75 in ever versus never breast fed (univariable)§ n=737	** **				0.68 (0.48 to 0.95)
OR (95% CI) CHAQ >0.75 in ever versus never breast fed (adjusted)¶ n=737					0.61 (0.39 to 0.95)

*Indicates variables included in multivariable logistic regression analysis.

†Indicates the number of children with the available measure when not present in all children.

‡JADAS-71: composite score of 71 active joint count, Physician Global Assessment, parent general evaluation and ESR.

§AUC 0.55.

¶AUC 0.84.

AUC, area under the curve; CHAQ, Childhood Health Assessment Questionnaire; ESR, erythrocyte sedimentation rate; ILAR, International League Against Rheumatism; JADAS, Juvenile Arthritis Disease Activity Score; JIA, juvenile idiopathic arthritis; Ref, reference; RF, rheumatoid factor; VAS, visual analogue scale.

**Table 2:** Demographic and clinical characteristics according to length of breast feeding*

Characteristic†	Never	<3 months	≥3 and <6 months	≥6 months	p Value for trend
N (%)	410 (46)	233 (26)	124 (14)	131 (15)	
Female, n (%)	262 (64)	154 (66)	85 (69)	90 (69)	0.2
Caucasian, n (%)	381 (93)	208 (89)	113 (91)	111 (85)	0.01
Index of Multiple Deprivation (n=737), n (%)					
Low	37 (11)	44 (23)	26 (27)	34 (32)	<0.001
Medium	139 (43)	93 (48)	45 (48)	47 (45)	
High	149 (46)	57 (29)	25 (25)	24 (23)	
Age at symptom onset (years), median (IQR)	7.5 (3.4, 11.5)	6.0 (2.5, 10.4)	5.0 (1.9,. 9.7)	4.8 (2, 9.9)	0.002
Symptom duration (months), median (IQR)	5.6 (2.8, 12.5)	5.5 (2.9, 10.3)	5.3 (2.9,. 14.3)	5.3 (2.9, 14.3)	0.5
Active joint count, median (IQR)	2 (1, 5)	2 (1,. 6)	2 (1, 4)	2 (1, 6)	0.4
Limited joint count, median (IQR)	1 (1, 3)	1 (1, 3)	1 (0, 3)	1 (1, 3)	0.08
Physician global assessment (n=743), median (IQR)	32 (18, 56)	30 (18, 57)	25 (12, 46)	24 (13, 49)	0.005
ESR (n=570), median (IQR)	18 (6, 47)	20 (6, 50)	29 (7, 55)	21 (9, 53)	0.1
CHAQ score (n=674), median (IQR)	0.88 (0.25, 1.63)	0.63 (0.13, 1.25)	0.63 (0.13, 1.25)	0.5 (0, 1.13)	<0.001
CHAQ >0.75 (n=674), n (%)	136 (46)	63 (39)	35 (41)	39 (37)	0.001
Parent general evaluation. (100 mm VAS) (n=656), median (IQR)	39 (10, 64)	16 (3, 45)	27 (4, 50)	17 (5, 42)	0.008
Pain (100 mm VAS) (n=664), median (IQR)	29 (7, 54)	22 (4, 53)	28 (5, 60)	20 (6, 45)	0.005
JADAS-71, median (IQR)	13.2 (7–19.9)	10.4 (5.9–16.9)	10.2 (5–21.3)	10.6 (4.5–15.6)	0.1
ILAR Subtype, n (%)					
Systemic arthritis	23 (6)	8 (4)	9 (7)	7 (5)	0.09
Oligoarthritis (persistent)	189 (46)	101 (43)	64 (51)	59 (45)	
Oligoarthritis (extended)	28 (7)	13 (6)	10 (8)	6 (4)	
Polyarthritis (RF negative)	79 (19)	73 (31)	22 (18)	38 (29)	
Polyarthritis (RF positive)	11 (3)	8 (3)	5 (4)	2 (2)	
Enthesitis-related arthritis	32 (8)	11 (5)	4 (3)	4 (3)	
Psoriatic arthritis	36 (9)	12 (5)	8 (7)	10 (8)	
Undifferentiated arthritis	12 (3)	7 (3)	2 (2)	5 (4)	

*Duration of breast feeding not reported for 25 children.

†Numbers in brackets in first column indicate available data where data items were missing or not available.

CHAQ, Childhood Health Assessment Questionnaire; ILAR, International League Against Rheumatism; JADAS, Juvenile Arthritis Disease Activity Scores; RF, rheumatoid factor.

The relationship between ever breast fed and CHAQ score was explored in 737 children resident in England. After adjustment, ever breast feeding was associated with a 39% lesser probability of presenting with a CHAQ >0.75 (OR 0.61 (95% CI 0.39 to 0.95)) (table 1).

## Discussion

This study represents the first JIA study to investigate the impact of breast feeding on presentation of disease, and found breast feeding was associated with an earlier but milder presentation, the latter of which persisted after adjusting for marked SES differences.

The observation of a younger age of onset is interesting and different from research focusing on coeliac disease, which suggests breast feeding may delay the onset of childhood coeliac disease,^7^ although this relationship is confounded by timing of gluten introduction. It has been suggested that breast milk may stimulate the immature immune system, thus, triggering the autoimmune process of JIA in the right setting.^8^ Earlier onset could also be explained by differences in ILAR categories observed between the two groups, with a higher proportion of PsA/ERA in the never-breastfed group, which tend to present at a later age.

Previous reports on breast feeding and JIA have largely excluded children with ERA/PsA. Why breast feeding might alter/change susceptibility to certain subtypes of JIA is not clear, although there have been reports of differences in the microbiome between children with ERA and those without.^9^ Breast feeding may have a protective effect on early intestinal infections, and contribute to constitution of the gut flora, but further research is required to validate these findings.

Children who were ever breast fed reported lower CHAQ, pain and PGE, which also followed a dose–response relationship. This could relate to differences observed among SES between the groups, which have been shown to associate with reporting of the severity of disease.^10^ However, the relationship between CHAQ and breast feeding persisted after adjustment for SES. T cells contribute an important role to the pathogenesis of JIA, and breast milk is hypothesised to have a T-cell specific suppressant effect, which may in turn, reduce severity of presentation.

Breastfeeding rates in our study were low (56%), but are in keeping with UK data, which highlights low UK rates compared with other European countries. It is possible that some misclassification of breastfeeding status is present as parents may have different views regarding when breast feeding becomes significant enough to answer the question ‘was your child ever breastfed?’ with ‘yes’. The fact that we have identified a link between breast feeding and presentation of JIA despite potential misclassification further strengthens our findings.

This study is not without limitations. We tried to capture SES using national deprivation scores, but these only refer to the area of residence and not the individuals themselves. They also represent the current SES and not necessarily that at the time of the child's birth. Unfortunately, data on maternal age and education were not available, but we did include referral hospital as a further geographical marker, which may influence the decision to breast feed. Data on exclusivity of breast feeding was not captured. Finally, and most importantly, as we did not have access to a control group, the data cannot be used to comment on susceptibility to JIA, including susceptibility to any individual ILAR subtype.

We recognise that these observations may have considerable impact on prospective parents, and therefore, it is important that these findings are explored further to consider how breast feeding might influence the presentation of JIA. Further research should also focus on whether breast feeding may influence the susceptibility to JIA and in particular, its different patterns.
